# Docetaxel, Oxaliplatin and Capecitabine (TEX) triplet regimen as adjuvant chemotherapy in resected gastric adenocarcinoma

**DOI:** 10.3332/ecancer.2021.1292

**Published:** 2021-09-21

**Authors:** Divya Bala Thumaty, Raju Titus Chacko, Ajoy Oommen John, Anjana Joel, Josh Thomas Georgy, Myla Jacob, Inian Samarasam, Dipti Masih, Rajesh Isaiah, Visalakshi Jeyaseelan, Ashish Singh

**Affiliations:** 1 Department of Medical Oncology, Christian Medical College, Ida Scudder Road, Vellore, TN, India; 2 Department of Upper GI surgery, Division of Surgery, Christian Medical College, Vellore, India; 3 Department of General Pathology, Christian Medical College, Vellore, India; 4 Department of Radiotherapy, Christian Medical College, Vellore, India; 5 Department of Biostatistics, Christian Medical College, Vellore, India

**Keywords:** gastric cancer, stomach cancer, FLOT, TEX, adjuvant chemotherapy

## Abstract

**Background:**

Adjuvant chemotherapy after surgery for gastric cancer improves survival but is difficult to administer due to poor tolerance. Combination chemotherapy with Docetaxel (Taxotere), Oxaliplatin (Eloxatin) and Capecitabine (Xeloda) (TEX) is used in the treatment of advanced gastric cancer. The efficacy and tolerability of this regimen (TEX) post resection of gastric cancer have not been studied.

**Materials and methods:**

Patients diagnosed with gastric adenocarcinoma, post resection without any prior chemotherapy between July 2007 and May 2011 and treated with TEX regimen administered as T 35 mg/m^2^ and E 50 mg/m^2^ on days (d) 1, 8 and X 625 mg/m^2^ bid (twice daily) on d 1–14 every 21 days were included in this retrospective analysis. Patient’s electronic medical records were studied and data on tolerance, progression‑free survival (PFS) and overall survival (OS) was collected.

**Results:**

Fifty-eight patients were treated with adjuvant TEX chemotherapy, majority 40 (68%) had distal gastric cancer. All patients underwent a D1 gastrectomy, and resection was performed for 44 (75%). Only 14 (24%) patients had more than 15 nodes studied in the resected specimen. Distribution for stages I, II and III is 14 (24%), 30 (52%) and 14 (24%), respectively. After a median follow-up of 40 months, the 3-year relapse free survival was 58% (95% CI: 42–68), and estimated median OS was 71 months (95% CI: 19–123 months). Twenty-three (40%) required dose reduction due to toxicity. Grade 3 or 4 toxicity was recorded for 22 (37%). Half (52%) of patients completed all planned chemotherapy of six cycles.

**Conclusion:**

Post resection of gastric adenocarcinoma adjuvant triplet TEX chemotherapy is a feasible and effective outpatient regimen. Diarrhoea, neutropenia and neuropathy were the common dose limiting toxicity. Post-surgery only half the numbers of patients are able to complete all planned cycles.

## Introduction

Gastric cancer tops the list of cancers associated with deaths and disability-adjusted life-years amongst all the cancers in the recent Global Burden of Disease Study of the burden and incidence of cancers in India [1].

Surgery followed by adjuvant chemotherapy with fluoropyrimidine with or without platinum is considered as a standard treatment for operable gastric cancers [2]. Docetaxel in combination with Oxaliplatin and 5 Fluorouracil has improved survival and has become the standard perioperative chemotherapy regimen in fit patients [3]. Perioperative approach can make systemic chemotherapy available to all patients. Preoperative chemotherapy however may not be possible in the presence of a bleeding ulcer or gastric outlet obstruction. Tolerance reported to adjuvant chemotherapy following gastrectomy is low with only 45%–60% of patient being able to start and complete all planned cycles following surgery [4, 5].

For advanced gastric cancers, one of the ways to make triplet chemotherapy more tolerable without compromising on efficacy is to replace 5Flurouracil (5FU) with Capecitabine and administer a lower dose of Oxaliplatin and Docetaxel on D1 and day 8 [6–9]. The efficacy of this combination when administered post operatively is not known. In the present study, we report the outcomes with use of Docetaxel, Oxaliplatin, Capecitabine (TEX) regimen in adjuvant setting at a single centre, in south India.

## Methods

This is a single centre, Institutional Review Board (IRB)-approved retrospective analysis of patients with gastric cancer who received TEX chemotherapy after surgery at our centre. All patients treated between May 2007 and April 2011 are included in this analysis. Patients satisfying all the following criteria were included in analysis: histologically proven gastric cancer who have undergone a complete resection (R0 or R1) and received at least one cycle of chemotherapy in our department, with no evidence of metastatic disease on CT scan of abdomen and chest imaging.

The regimen consisted of Docetaxel administered at 35 mg/m^2^ on day 1 and day 8, Oxaliplatin at 50 mg/m^2^ on days 1 and 8 and Capecitabine at 1,250 mg/m^2^/day, days 1–14, cycle repeated every 21 days. Toxicity assessment was done at every patient visit and recorded as per NCI–CTCAE version 4.0. End points assessed were relapse free survival defined as time from diagnosis to relapse or death and overall survival (OS) defined as time from diagnosis to death.

### Clinical data collection and statistics

For this study, demographic data and baseline clinical data were collected retrospectively from the electronic medical records and patients were contacted by telephone regarding the disease status and survival. Follow-up data was updated till September 2019. All data were entered in SPSS Statistics version 21 (IBM) and used for analysis. Descriptive statistics including median, frequency and percentage for categorical variables was used to describe age, gender distribution, treatment and response to treatment. Median event-free survival (EFS) and OS were calculated using Kaplan–Meier estimates.

## Results

Of all the inpatient records reviewed between May 2007 and April 2011, 384 patients were admitted for administration of chemotherapy for gastric adenocarcinoma (including adjuvant, neoadjuvant and metastatic disease). Adjuvant chemotherapy was administered to 93 patients, of which 18 patients received only a doublet chemotherapy with Oxaliplatin and 5-Flurouracil while 75 patients received TEX. Fifty-eight patients who fulfilled the inclusion criterion with available date were included in this retrospective study. Patient and disease characteristics are presented in Table 1. Median age at presentation was 53 years (range: 30–70 years). Gastric cancer as expected was commoner in men (69%), and majority (77%) had distal tumours. Patients with proximal tumour were more likely to receive neoadjuvant chemotherapy and therefore represent only 22% in this study.

Surgical findings and final pathological stage are presented in Table 2. Staging was I, II and III in 14 (24%), 30 (52%) and 14 (24%), respectively; There were four patients with stage 1a tumour who received chemotherapy – indications being – distal margin positivity and lymphovascular invasion. Forty-four (76%) patients had <15 nodes harvested from the surgical specimen.

**Figure 1.**
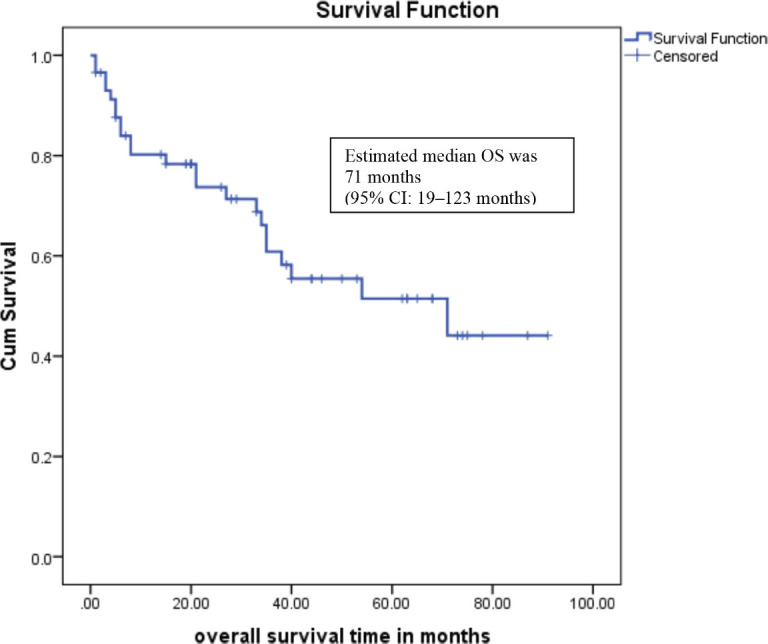
Overall survival.

The median time to start of adjuvant chemotherapy was 3 weeks. After a median follow-up of 40 months, the 3-year relapse free survival was 58% (95% CI: 42–68), and estimated median OS was 71 months (95% CI: 19–123 months) as presented in Figure 1*.*

Toxicity recorded is presented in Table 3.

Twenty-three (40%) required dose reduction due to toxicity. Grade 3 or 4 toxicity was recorded for 22 (37%). 52% of patients completed all planned chemotherapy. Ten (17%) patients discontinued chemotherapy due to grade 3 toxicity.

## Discussion

About two decades ago, the intergroup 0116 trial reported improved survival with adjuvant chemoirradiation post resection for gastric adenocarcinoma [10]. Locoregional relapse reduction accounted for majority of overall relapse reduction [11]. Since then, trials have confirmed that after a radical resection for gastric cancer and adjuvant chemotherapy, addition of chemo irradiation does not improve survival [5, 12, 13]. Oral chemotherapy with S1 or Capecitabine as a single agent or combination with Oxaliplatin is effective and is the standard treatment post resection [14, 15]. In another study for early stage gastric cancers, Epirubicin when combined with Cisplatin and Capecitabine did not improve outcomes over the doublet alone [16]. We designed the TEX adjuvant regimen based on the findings from published reports that showed addition of Docetaxel to platinum and 5FU resulted in better response rates and PFS in multiple reports [7, 8].

The assumption was Docetaxel may be better than Epirubicin in early gastric cancer. Later on it was shown that Docetaxel in combination with Oxaliplatin and infusional 5 Fluorouracil (FLOT) was superior to Epirubicin, Cisplatin, Flurouracil (ECF) in patients receiving perioperative chemotherapy for non-metastatic gastric cancer [3]. A triplet regimen like FLOT or TEX is expected to improve outcomes, provided it can be administered safely to patients post major surgery like gastrectomy. Administration of chemotherapy post-surgery is poorly tolerated with only 45%–60% of patients able to start chemotherapy after surgery and less number are able to complete the entire planned therapy [4, 5] Replacing 5-FU with Capecitabine obviates the need for an infusion pump and central line, associated with increase in cost and catheter related complications, both drugs have a similar incidence of toxicities, though a different profile. TEX is easy to administer as an outpatient treatment as compared to FLOT. Postoperatively more than six cycles are expected to be poorly tolerated due to neuropathy, hand foot syndrome and diarrhoea.

Seventy-eight percent of patients had a distal cancer; upfront surgery is more common for distal cancers for reasons such as obstruction or bleeding. Perioperative chemotherapy is preferred for proximal lesions in the absence of contraindications for chemotherapy. Surgery performed was not D2 resection for any of our patients. However, D1 or an extended D1 gastrectomy is associated with lesser morbidity without any significant compromise on survival [17]. 12 (21%) patients had R1 resection. Adjuvant chemo irradiation is poorly tolerated and is not standard of care at our centre. Our results indicate that TEX is feasible and is an effective regimen with 58% of patients being free of disease recurrence at 3 years after diagnosis. These results are despite the fact that 48% of patients were unable to complete all assigned therapy. The median OS was 71 months, higher as compared to the published literature. This is probably due to the inclusion of only fit patients and early stage disease (stage 1, 2 amounting to 77% of patients) who are able to start chemotherapy and a smaller sample size.

Most relapses were distant, and data on local only relapse rate is not available.

Fatigue, diarrhoea, hand foot syndrome, neutropenia and neuropathy were the major toxicity with this regimen. There was one treatment related death due to febrile neutropenia and septic shock that occurred soon after the first dose. Dihydropyrimidine dehydrogenase (DPD) enzyme deficiency testing may predict such severe toxicity with fluoropyrimidines but isn’t yet done for every patient at our centre. Cumulative toxicities like hand foot syndrome, fatigue and neuropathy developed over time and were the main reason for dose reduction and early discontinuation of treatment. From this experience, we suggest that a modification in the schedule which allows infusion of Oxaliplatin and Docetaxel every biweekly in order to reduce toxicity and make the regimen tolerable to more patients who are at risk of relapse.

The limitations of our study include the retrospective nature, absence of a control arm and inclusion of only patients who were able to start chemotherapy after surgery.

## Conclusion

Post resection of gastric adenocarcinoma adjuvant TEX is a feasible outpatient triplet chemotherapy regimen with acceptable results. Diarrhoea, neutropenia and neuropathy are the common toxicity requiring dose adjustments. Shortening the duration of adjuvant treatment by use as perioperative treatment may reduce toxicity, improve compliance and should be studied.

## Conflicts of interest

Nil.

## Funding

Source(s) of support: CMC Fluid Research Grant.

## Figures and Tables

**Table 1.** Baseline characteristics.

Patient and tumour characteristic	Total *n* = 58 (%)
Age (Median, range)	53 years, Range: 30–70 years
Gender Males Females	40 (68%) 18 (31%)
Site Distal Proximal	45 (78%) 13 (22%)
Histology Poorly differentiated Moderately differentiated Well differentiated	29 (50%) 26 (45%) 3 (5%)
Signet ring histology	12/58 (20%)
LVI present	31/58 (53%)
PNI present	19/58 (32%)

LVI: lymphovascular invasion; PNI: perineural invasion

**Table 2.** Surgical details

Surgical details	Number (%)
Type of gastrectomy Total Subtotal Distal	*N* = 58 13 (22%) 38 (65%) 7 (12%)
Lymph node dissection D1 D1 + D2 D0	*n* = 5411 (20%) 42 (77%) 0 1
No of lymph node dissected <15 ≥15	*n* = 57 43 14
Completeness of resection R0 R1 R2 Not known	*N* = 58 44 12 0 2
Final path TNM stage 1 2 3 4	*N* = 58 14 30 14 0

**Table 3.** Adverse events

Toxicity	Grade 1–2, *n* (%)	Grade 3–4, *n* = 22 (37%)
Anaemia	20 (34%)	6 (10%)
Thrombocytopenia	14 (24%)	4 (6%)
Febrile neutropenia	2 (3%)	4 (6%)
Diarrhoea	16 (28%)	8 (13%)
Neuropathy	12 (20%)	5 (9%)
Hand foot syndrome	24 (41%)	6 (10%)
Hypersensitivity	3 (5%)	1 (2%)
